# Kinetics of lithium peroxide oxidation by redox mediators and consequences for the lithium–oxygen cell

**DOI:** 10.1038/s41467-018-03204-0

**Published:** 2018-02-22

**Authors:** Yuhui Chen, Xiangwen Gao, Lee R. Johnson, Peter G. Bruce

**Affiliations:** 1 Departments of Materials and Chemistry, University of Oxford, Parks Road, Oxford, OX1 3PH UK; 2 School of Chemistry and GSK Carbon Neutral Laboratory for Sustainable Chemistry, University of Nottingham, Jubilee Campus, Nottingham, NG7 2TU UK

## Abstract

Lithium–oxygen cells, in which lithium peroxide forms in solution rather than on the electrode surface, can sustain relatively high cycling rates but require redox mediators to charge. The mediators are oxidised at the electrode surface and then oxidise lithium peroxide stored in the cathode. The kinetics of lithium peroxide oxidation has received almost no attention and yet is crucial for the operation of the lithium–oxygen cell. It is essential that the molecules oxidise lithium peroxide sufficiently rapidly to sustain fast charging. Here, we investigate the kinetics of lithium peroxide oxidation by several different classes of redox mediators. We show that the reaction is not a simple outer-sphere electron transfer and that the steric structure of the mediator molecule plays an important role. The fastest mediator studied could sustain a charging current of up to 1.9 A cm^–2^, based on a model for a porous electrode described here.

## Abstract (web summary)

The kinetics of Li_2_O_2_ oxidation is of high importance to the operation of Li–O_2_ batteries. Here the authors work on different types of mediators revealing the dependence of the kinetics on the nature of the redox active site and its steric hindrance.

## Introduction

The rechargeable aprotic lithium–O_2_ (air) battery operates by the reduction of O_2_ at the positive electrode forming Li_2_O_2_ on discharge, with oxidation of Li_2_O_2_ taking place on charge^1–10^. Li_2_O_2_ is an insulating and insoluble solid^11–16^. Ether-based electrolytes, such as dimethoxyethane (DME) and tetra ethylene glycol dimethyl ether (tetraglyme), have been used as the basis of electrolyte solutions in most Li–O_2_ cells, because of their relative stability towards reduced oxygen species. However, they cannot dissolve LiO_2_, the intermediate in the reduction of O_2_ to Li_2_O_2_,

1 $${\rm O}_2 + {\rm L}{\rm i}^ + + {\rm e}^- \to {\rm LiO}_2$$

2 $$2{\rm LiO}_2 \to {\rm Li}_2{\rm O}_2 + {\rm O}_2$$

3 $${\rm LiO}_2 + {\rm Li}^ + + {\rm e}^- \to {\rm Li}_2{\rm O}_2$$

resulting in LiO_2_ being adsorbed on the electrode surface, and resulting in the growth of Li_2_O_2_ films on the electrode, leading to low rates, low capacities and early cell death^14,17^. The problem is exacerbated by the formation of Li_2_CO_3_ between Li_2_O_2_ and carbon, the latter is usually employed as the material for the porous positive electrode^18^. Use of redox mediators (RMs) on discharge, such as 2,5-di-tert-butyl-1,4-benzoquinone (DBBQ), which are reduced at the electrode surface on discharge and then go on to reduce O_2_ to Li_2_O_2_ in solution, can help to mitigate these problems, but result in the formation of Li_2_O_2_ disconnected from the electrode surface and therefore electronically isolated during charging^19^. This introduces the need for a redox mediator to be employed on charging that can oxidise Li_2_O_2_^20–33^. Such mediators are molecules capable of oxidation at the surface of the pores in the porous positive electrode on charging and then transfer of holes to the electronically isolated Li_2_O_2_ particles within the pores. As a result, Li_2_O_2_ is oxidised and O_2_ released, the mediator molecule being reduced in the process and returning to the electrode surface for the cycle to be repeated.

Suitable oxidation mediators must have a redox potential above that for O_2_/Li_2_O_2_: 2.96 V vs. Li^+^/Li, a sufficiently high heterogeneous rate constant for electron transfer at the electrode surface to support the required charging rates, a highly reversible redox process such that the cycle may be carried out many times, and of course not only be capable of oxidising Li_2_O_2_, but with a sufficiently high rate to sustain the required charging current^34^. Stability of the mediators, especially on long-term cycling, is also an important challenge and recent work has considered the design of more stable redox mediators for cycling^22^; however, very little is known about the factors affecting the reaction between oxidation mediators and Li_2_O_2_. It is often assumed that a mediator with a high redox potential has fast kinetics for the oxidation of Li_2_O_2_, but this is not necessarily so^33^. Alternatively, the kinetics of Li_2_O_2_ oxidation by a mediator could be linked to the kinetics of its own redox process, but this would only be the case if both were outer-sphere electron transfer processes. Importantly, little experimental evidence exists about the kinetics of Li_2_O_2_ oxidation by redox mediators, yet their use and such kinetics are crucial to the operation of the Li–O_2_ cell.

Here, we investigate the kinetics of Li_2_O_2_ oxidation by several classes of redox mediators, which differs in the *E*^o^ (standard redox potential) and *k*^o^ (standard heterogeneous electron transfer rate constant) values, to ascertain the factors that control the rate of Li_2_O_2_ oxidation by the mediators.

## Results

### Apparent rate constants (*k*_app_) of mediators

Apparent rate constants (*k*_app_) for Li_2_O_2_ oxidation by the redox mediators were determined using scanning electrochemical microscopy (SECM). Details of the cell and procedures used are given in the Methods section. In brief, SECM feedback approach curves at a Li_2_O_2_ disk, composed of a pressed pellet of commercial Li_2_O_2_ with a diameter of 12 mm, were recorded and apparent rate constants, *k*_app_, for Li_2_O_2_ oxidation were obtained by fitting to the theoretical feedback approach curves developed by Cornut et al.^35–37^. When recording an approach curve, the SECM tip was held at a sufficiently positive potential such that a steady-state current was obtained for the oxidation of the redox mediator. The tip approaches the Li_2_O_2_ disk and at small separation distances, the mediator oxidised at the tip diffuses to the Li_2_O_2_ disk where it oxidises Li_2_O_2_, regenerating itself and contributing to a feedback loop, while concurrently, diffusion of the mediator to the tip is blocked by the surface. The balance of the two alter the current at the SECM tip, *i*_T_, and the faster the kinetics of Li_2_O_2_ oxidation by the mediator the greater the current, see Supplementary Figure 1. As we do not know the mechanism by which the mediators oxidise the lithium peroxides surface, we can only obtain an apparent rate constant (*k*_app_) based on the feedback response; however, this provides a comparison between the different mediators and indicates the overall rate capability.

**Fig. 1:**
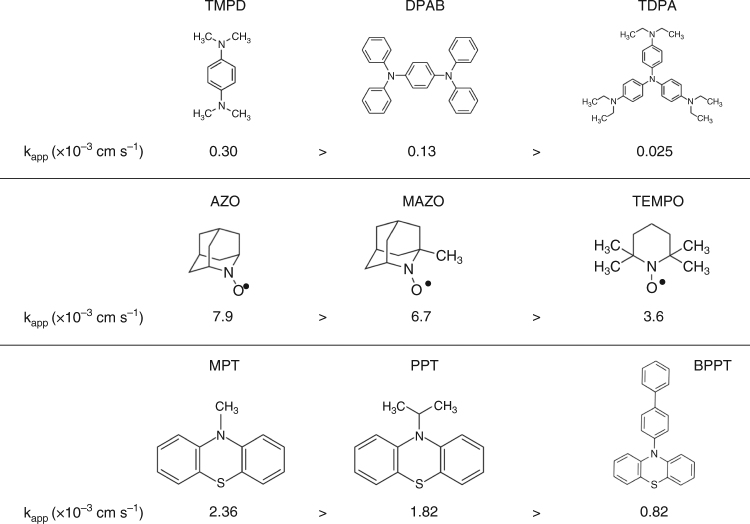
Structures of the oxidation mediators and their kinetics of Li_2_O_2_ oxidation. Comparison of the apparent rate constants (*k*_app_) for the reaction between the redox mediators and Li_2_O_2_ grouped by structure

Fig. 1 shows the oxidation mediators studied. They are in three classes, amines, nitroxy and thiol compounds, chosen because they are classes of compounds known to exhibit reversible redox processes and include several of the compounds that have been used as oxidation mediators in Li–O_2_ cells, such as tris[4-(diethylamino)phenyl]amine (TDPA), 2,2,6,6-tetramethyl-1-piperidinyloxy (TEMPO) and 10-methylphenothiazine (MPT)^20–22^. *k*_app_ for Li_2_O_2_ oxidation by the mediators are presented in Supplementary Table 1. The standard redox potential, *E*^o^, and standard heterogeneous electron transfer rate constant, *k*^o^, were measured for each mediator using cyclic voltammetry, as described in the Methods section. The diffusion coefficients, *D*, were obtained from the steady-state current at an ultramicroelectrode (UME), also as described in the Methods section. The values for each of the three parameters are also given in Supplementary Table 1. Three additional mediators, tetrathiafulvalene (TTF), ferrocene (FC) and 5,10-dimethylphenazine (DMPZ), which do not belong to the above three classed, but have been commonly used as oxidation mediators, were also studied and are listed in Supplementary Table 1^23,24,38^. The standard redox potentials are all positive for the O_2_/Li_2_O_2_ reaction. The diffusion coefficients vary by no more than a factor of 3. The *k*^o^ for the mediators themselves are all relatively high, ranging from 0.007 to 0.078 cm s^–1^, sufficiently so to support an areal current density over 200 mA cm^−2^ at an overpotential of 60 mV, based on the true surface area of the pores and therefore more than sufficient to sustain an areal current density suitable for a Li–O_2_ cell. Of course, this does not take into account the kinetics of Li_2_O_2_ oxidation required to sustain the current, which will be discussed below after the presentation of the rates of Li_2_O_2_ oxidation. The assumptions regarding the porous cathode structure and the approach used to make this estimate are described in the Supplementary Note.

**Fig. 2:**
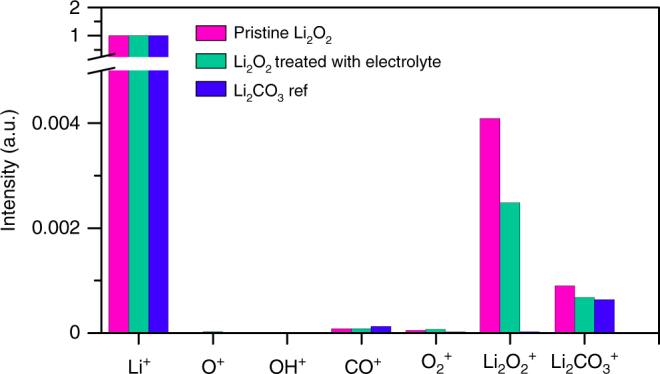
TOF-SIMS of Li_2_O_2_ disks before and after treating with electrolyte of 1 M LiTFSI in tetraglyme. Both Li_2_O_2_ disks show signal of Li_2_O_2_^+^ and Li_2_CO_3_^+^ whereas the Li_2_CO_3_ disk shows little signal of Li_2_O_2_^+^, confirming the presence of Li_2_O_2_ on the surface of disk after treating with the electrolyte

Before considering the kinetics of the mediator oxidation in more detail, we first determine the surface composition of the disk and the possibility of passivation with, for example, Li_2_CO_3_. A disk of Li_2_O_2_ was immersed in 1 M LiTFSI in tetraglyme for 3 h and then examined by time of flight secondary ion mass spectrometry (TOF-SIMS), alongside a disk that was not exposed to the electrolyte solution. As shown in Fig. 2, for both disks, the major peaks are from Li_2_O_2_^+^, with the secondary peaks being ascribed to Li_2_CO_3_. These results show that although there is some Li_2_CO_3_, even on the surface of the pristine disk, a significant proportion of the surface remains as Li_2_O_2_ even after 3 h of exposure to the electrolyte, confirming that the disk is suitable for the SECM measurements. Note that the sensitivity of TOF-SIMS to different species varies, consequently it is not possible to quantify the relative amounts of Li_2_O_2_ and Li_2_CO_3_ by simply comparing the areas under the peaks. Instead, the disk was etched until the signal from Li_2_O_2_ was constant, therefore corresponding to the bulk peroxide, i.e., 100% Li_2_O_2_. Comparing this signal with that for Li_2_O_2_ at the surface indicated that approximately 35% of the disk surface was Li_2_O_2_.

A disk of Li_2_CO_3_ was investigated with SECM using TEMPO as the oxidation mediator, as it has a sufficiently high potential to oxidise Li_2_CO_3_ and shows fast kinetics with the Li_2_O_2_ disk. The results are shown in Supplementary Figure 2. The *k*_app_ for oxidation of Li_2_CO_3_ by TEMPO is four orders of magnitude lower than the data collected on the Li_2_O_2_ disk, indicating that even for mediators with sufficiently high potentials the contribution of Li_2_CO_3_ oxidation to the *k*_app_ is very small. The dominant reaction for the range of mediators studied here, even taking account of partial coverage by Li_2_CO_3_, is oxidation of Li_2_O_2_.

It has been reported previously by us and by others that several of the redox mediators used in Li–O_2_ cells to date exhibit some degree of decomposition^24,39–41^. Assembling a cell with commercial Li_2_O_2_ and the oxidation mediators TTF and AZO, and then charging to a capacity of ∼1 mAh results in notable decomposition of TTF and AZO as seen by ^1^HNMR of the electrolyte, see Supplementary Figure 3. In the SECM experiments, only a small amount of charge, ∼1 nAh, is passed, therefore the fraction of mediator that is decomposed is negligible.

### Inner-sphere process for mediator oxidising Li_2_O_2_

**Fig. 3:**
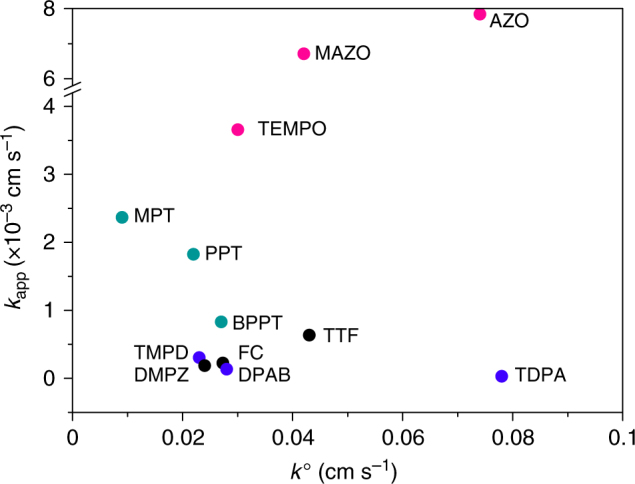
Dependence of the apparent rate constant, *k*_app_, on the heterogeneous electron transfer rate constant, *k*^o^, of the mediators. Amines, nitroxy and thiol compounds are marked in blue, red and green

**Fig. 4:**
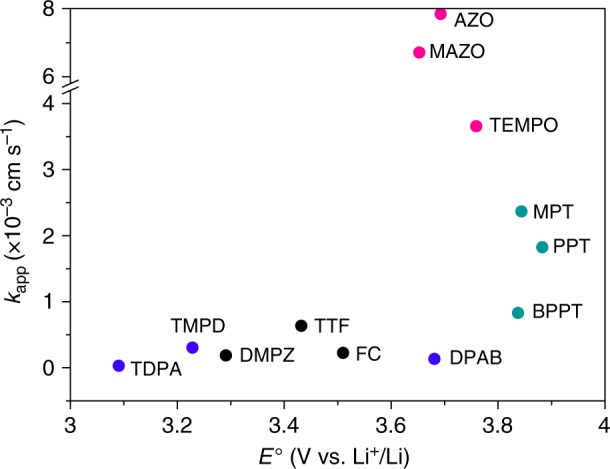
Dependence of the apparent rate constant, *k*_app_, on the redox potential, *E*^o^, of the mediators. Amines, nitroxy and thiol compounds are marked in blue, red and green

To explore the possible correlations between *k*_app_ and the electrochemical parameters of the redox mediators, *E*^o^ and *k*^o^, plots of *k*_app_ vs. *k*^o^ and *E*^o^ and are presented in Figs. 3 and 4, respectively. There is no apparent dependence of *k*_app_ on *k*^o^, Fig. 3. The values of k^o^ for the different redox mediators appear independent of the nature of the electrode used to measure them, as demonstrated by measuring these values at Au and glassy carbon electrodes, see Supplementary Figure 4 and Methods section, consistent with the RM^+^/RM reactions occurring by outer-sphere electron transfer. If the oxidation of Li_2_O_2_ was also an outer-sphere electron transfer reaction, then *k*_app_ would be proportional to *k*^o^ of the redox mediator (and hence the reorganisation energy of the RM and surrounding solution), or the rate of the reaction Li_2_O_2_ → Li_2_O_2_^+^ + e^−^. Since there is no dependence of *k*_app_ on *k*^o^, the former cannot be true. If the rate was limited by the electron transfer kinetics associated with the Li_2_O_2_, then *k*_app_ would be invariant, which again is not the case. We conclude that oxidation of Li_2_O_2_ by the redox mediators is mainly an inner-sphere process, i.e., involves adsorption of the mediator on the peroxide surface. The values for *k*_app_ are one order of magnitude smaller than the corresponding *k*^o^ values, indicating that the reaction of mediators oxidising Li_2_O_2_ is most likely to be the rate determining step of the entire charge process. This will particularly be true towards the end of charge when the surface area of the remaining Li_2_O_2_ is low. We estimate that a *k*_app_ from 2.5 × 10^–5^ to 7.9 × 10^–3^ cm s^–1^ in a Li–O_2_ cell with a porous cathode filled with Li_2_O_2_ would provide an areal current density of 108 mA cm^–2^ to 1.9 A cm^–2^ using the same model for the porous cathode as above. The details are described in the Supplementary Note and Supplementary Figure 5. Although we note that this equivalent charging current varies with consumption of Li_2_O_2_, it is sufficient to sustain the charging process, even for some of the slowest oxidation mediators investigated here.

Turning to the plot of *k*_app_ vs. *E*^o^, Fig. 4, it appears that the highest rates are observed for mediators with potentials above ∼3.6 V. However, potential per se is not the explanation for the high rate, as there are examples of mediators with a high potential but low rate, e.g., BPPT. From the experiment on the Li_2_CO_3_ disk using TEMPO, we know higher rates at high potentials are not due to the onset of Li_2_CO_3_ oxidation contributing to the overall surface oxidation kinetics. Different crystal facets of Li_2_O_2_ will have different oxidation potentials^42^. Mediators operating at higher potentials could oxidise these higher potential facets and hence access a greater Li_2_O_2_ surface area. However, the fact that the rates vary for different mediators above 3.6 V and several high potential mediators have relatively low *k*_app_ suggests that this alone cannot be the reason for high rate mediators having a relatively high potential. As discussed below, we believe an important factor controlling the rate of the mediators is the nature of the oxidising centre and the degree of its steric hindrance.

Considering the molecules presented in Fig. 1 and the *k*_app_ values shown in the figure, it is evident that the nitroxy radicals exhibit the fastest rates of Li_2_O_2_ oxidation. The thiol group also provides a high rate, in contrast to the amines that are all low rate. The chemistry of the redox centre appears to be an important factor for controlling the rate of oxidation, probably due to the interaction with Li_2_O_2_ surface. The oxidation rates decrease when the redox centre of the molecule is surrounded by bulky groups, Fig. 1. This suggests that a key factor influencing the kinetics of Li_2_O_2_ oxidation is the steric hindrance as the molecule approaches the surface of Li_2_O_2_. The fastest kinetics is exhibited by 2-azaadamantane-*N*-oxyl (AZO), 7.9 × 10^–3^ cm s^–1^, which has the most exposed redox centre of all the redox mediators studied here. This observation is in accord with the lack of evidence for an outer-sphere reaction and provides direct evidence for Li_2_O_2_ oxidation proceeding by an inner-sphere mechanism.

## Discussion

In conclusion, we have measured the rate constants for the oxidation of Li_2_O_2_ particles by a series of molecular mediators spanning standard redox potentials, *E*^o^ from 3.1 to 3.9 V and standard heterogeneous rate constants for electron transfer, *k*^o^ from 0.007 to 0.078 × 10^–3^ cm s^–1^. The surface of Li_2_O_2_ particles in a typical electrolyte solution, LiTFSI in tetraglyme, is partially covered by Li_2_CO_3_, but the rate of Li_2_CO_3_ oxidation, a mediator that operates at 3.8 V, TEMPO, is four orders of magnitude lower than for Li_2_O_2_, therefore Li_2_O_2_ oxidation dominates. There is no correlation between the variation of *k*^o^, the standard heterogeneous rate constant at the electrode surface for the mediators, and the rate of Li_2_O_2_ oxidation by the mediators, indicative of this not being an outer-sphere electron transfer process at the Li_2_O_2_ surface. There is evidence of Li_2_O_2_ oxidation rates depending on the nature of the oxidising molecule. Nitroxy radicals, especially those with low steric hindrances of access to the Li_2_O_2_ surface, exhibit the highest rates. Nevertheless, the mechanism of Li_2_O_2_ oxidation by molecular oxidants is still not well understood, and such understanding will be important in order to inform the design of optimised oxidation mediators. All mediators studied display kinetics sufficient to enable relatively high rates within a battery, charging current density exceeding 100 mA cm^–2^. A mediator with a *k*_app_ of 7.9 × 10^–3^ cm s^–1^ can sustain an areal current density of up to 1.9 A cm^–2^, based on the same model. It is important to note that stability is still a challenge for the Li–O_2_ battery and here we observe significant mediator decomposition when passing large amounts of charge. More stable electrolytes and mediators are required to minimise side reactions and hence improve cycleability.

## Methods

### Materials preparation

Li_2_O_2_ and Li_2_CO_3_ disks were obtained by pressing Li_2_O_2_ powder (Aldrich) and Li_2_CO_3_ powder (Aldrich) with a die set in an Ar-filled glove box. Disks of 13 mm diameter and ∼1 mm of thickness were prepared and served as substrate. A Au microelectrode (diam. 25 μm, CHI) served as an SECM probe tip. Prior to measurement, the Au tip was polished with a microelectrode beveller (Sutter) and checked with a microscope. A silver wire reference electrode (RE) and a platinum counter electrode (CE) were used. 2,2,6,6-tetramethyl-1-piperidinyloxy (TEMPO), 2-azaadamantane-*N*-oxyl (AZO), 1-methyl-2-azaadamantane-*N*-oxyl (MAZO), tris[4-(diethylamino)phenyl]amine (TDPA), 1,4-bis(diphenylamino)benzene (DPAB), *N*,*N*,*N*′,*N*′-tetramethyl-p-phenylenediamine (TMPD), 10-methylphenothiazine (MPT), 10-isopropylphenothiazine (PPT), 10-(4-biphenylyl)phenothiazine (BPPT), tetrathiafulvalene (TTF), ferrocene (FC) and 5,10-dimethylphenazine (DMPZ) are from Aldrich. 10 mM redox mediators are dissolved in 100 mM LiTFSI–tetraglyme electrolyte for electrolyte solution.

A Swagelok cell was assembled as reported previously^40^, using a piece of gas diffusion layer electrode (GDL) as the positive electrode. A lithium super ionic conductor disc (LiSICON, Ohara) was used to protect Li metal as the negative electrode. A Li_2_O_2_ disk was placed between the GDL and the LiSICON essentially placing the cell in a discharged state. TTF and AZO were chosen as the oxidation mediators. The cell was charged by holding at 3.4 V for TTF and 3.7 V for AZO until 1 mAh charge passed prior to further chemical characterisations. For NMR analysis, the electrodes and separators were rinsed with 0.7 ml of CDCl_3_, and measurements were recorded on a Bruker spectrometer (400 MHz).

### Electrochemical measurements

SECM experiments were performed with SECM bipotentiostat (CHI 920) in an Ar-filled glovebox. Prior to kinetics measurement, the NG factor of Au tip was determined by approaching a completely insulating surface and fitting the negative approach curve. The data processing and fitting process were described elsewhere^35–37^. A dimensionless rate constant, *κ*, was obtained by data fit, which equals to *k*_app_ *r*_o_/*D*, where *r*_0_ is the radius of tip and *D* is the diffusion coefficient of redox mediators. *D* of various mediators were determined by measure steady-state current of a Au microelectrode with known radius *r*_0_, according to *i*_ss_=4*nFDr*_o_*C*.

The redox potential and heterogeneous electron transfer rate constants *k*^o^ of redox mediators itself were determined using cyclic voltammetry(CV) measurements. The redox potential is determined by the centre of two redox peaks, which is measured in a 100-mM LiTFSI–tetraglyme solution with 10 mM of various mediators at a Au electrode. Partially charged LiFeO_4_ (LFP) protected by a glass frit served as an RE and it gave a potential of 3.45 V vs. Li^+^/Li as reported previously. A platinum wire served as a CE. The details of *k*^o^ measurement are described elsewhere^43^. Briefly, CVs were recorded at various scan rates, ranging from 0.05 to 10 V s^–1^. *Ψ*, a function of CV peaks separation, was plotted vs. root of scan rate and a linear fit was applied. *k*^o^ was obtained from the slope of linear fit. The *k*^o^ measurement was carried out at both Au and glassy carbon (GC) WEs. Due to the non-negligible resistance of ether-based electrolytes, an Ohmic overpotential correction was applied to account for the uncompensated resistence during  CV measurements and a silver wire RE was used.

### Characterisations

For the surface characterisations, the Li_2_O_2_ disk was immersed in 1 M LiTFSI–tetraglyme solution for 3 h prior to XPS and TOF-SIMS experiments. Both pristine disk and treated disk were characterised in an air-sensitive holder. To measure the TOF-SIMS of bulk Li_2_O_2_, the data were recorded after 2 min etching.

### Data availability

The data that support the findings of this study are available from the corresponding author upon reasonable request. Background data has been deposited in the Oxford Research Archive (ORA) at: https://ora.ox.ac.uk/objects/uuid:c23a0cc0-55b5-455f-bb68-a14d8ea2e3bc.

## Electronic supplementary material

Supplementary Information

## References

[1] Aurbach D, McCloskey BD, Nazar LF, Bruce PG (2016). Advances in understanding mechanisms underpinning lithium–air batteries. Nat. Energy.

[2] Abraham KM (2015). Prospects and limits of energy storage in batteries. J. Phys. Chem. Lett..

[3] Lu J (2014). Aprotic and aqueous Li–O_2_ batteries. Chem. Rev..

[4] Gallagher KG (2014). Quantifying the promise of lithium–air batteries for electric vehicles. Energy Environ. Sci..

[5] Luntz AC, McCloskey BD (2014). Nonaqueous Li–air batteries: a status report. Chem. Rev..

[6] Black R, Adams B, Nazar LF (2012). Non–aqueous and hybrid Li–O_2_ batteries. Adv. Energy Mater..

[7] Choi JW, Aurbach D (2016). Promise and reality of post–lithium–ion batteries with high energy densities. Nat. Rev. Mater..

[8] Imanishi, N., Luntz, A. C. & Bruce, P. G. *The Lithium Air Battery: Fundamentals* (Springer, New York, 2014).

[9] Feng N, He P, Zhou H (2016). Critical challenges in rechargeable aprotic Li–_O2_batteries. Adv. Energy Mater..

[10] Galloway TA, Hardwick LJ (2016). Utilizing in situ electrochemical SHINERS for oxygen reduction reaction studies in aprotic electrolytes. J. Phys. Chem. Lett..

[11] Viswanathan V (2011). Electrical conductivity in Li_2_O_2_ and its role in determining capacity limitations in non–aqueous Li–O_2_ batteries. J. Chem. Phys..

[12] Gerbig O, Merkle R, Maier J (2013). Electron and ion transport in Li_2_O_2_. Adv. Mater..

[13] Luntz AC (2013). Tunneling and polaron charge transport through Li_2_O_2_ in Li–O_2_ batteries. J. Phys. Chem. Lett..

[14] Johnson L (2014). The role of LiO_2_ solubility in O_2_ reduction in aprotic solvents and its consequences for Li–O_2_batteries. Nat. Chem..

[15] Kowalczk I, Read J, Salomon M (2007). Li–air batteries: a classic example of limitations owing to solubilities. Pure Appl. Chem..

[16] Wang J, Zhang Y, Guo L, Wang E, Peng Z (2016). Identifying reactive sites and transport limitations of oxygen reactions in aprotic lithium–O_2_ batteries at the stage of sudden death. Angew. Chem. Int. Ed..

[17] Burke CM, Pande V, Khetan A, Viswanathan V, McCloskey BD (2015). Enhancing electrochemical intermediate solvation through electrolyte anion selection to increase nonaqueous Li–O_2_ battery capacity. Proc. Natl Acad. Sci. USA.

[18] McCloskey BD (2012). Twin problems of interfacial carbonate formation in nonaqueous Li–O_2_ batteries. J. Phys. Chem. Lett..

[19] Gao X, Chen Y, Johnson L, Bruce PG (2016). Promoting solution phase discharge in Li–O_2_ batteries containing weakly solvating electrolyte solutions. Nat. Mater..

[20] Kundu D, Black R, Adams B, Nazar LF (2015). A highly active low voltage redox mediator for enhanced rechargeability of Lithium–oxygen batteries. ACS Cent. Sci..

[21] Bergner BJ, Schurmann A, Peppler K, Garsuch A, Janek J (2014). TEMPO: a mobile catalyst for rechargeable Li–O_2_ batteries. J. Am. Chem. Soc..

[22] Feng N, Mu X, Zhang X, He P, Zhou H (2017). Intensive study on the catalytical behavior of N–methylphenothiazine as a soluble mediator to oxidize the Li_2_O_2_ cathode of the Li–O_2_ battery. ACS Appl. Mater. Interfaces.

[23] Chen Y, Freunberger SA, Peng Z, Fontaine O, Bruce PG (2013). Charging a Li–O_2_ battery using a redox mediator. Nat. Chem..

[24] Lim HndashD (2016). Rational design of redox mediators for advanced Li–O_2_batteries. Nat. Energy.

[25] Bergner BJ (2015). Understanding the fundamentals of redox mediators in Li–O_2_ batteries: a case study on nitroxides. Phys. Chem. Chem. Phys..

[26] Sun D (2014). A solution–phase bifunctional catalyst for lithium–oxygen batteries. J. Am. Chem. Soc..

[27] Lim HndashD (2014). Superior rechargeability and efficiency of lithium–oxygen batteries: hierarchical air electrode architecture combined with a soluble catalyst. Angew. Chem. Int. Ed..

[28] Kwak WndashJ (2016). Li–O_2_ cells with LiBr as an electrolyte and a redox mediator. Energy Environ. Sci..

[29] Kwak WndashJ (2015). Understanding the behavior of Li–oxygen cells containing LiI. J. Mater. Chem. A.

[30] Liang Z, Lu YC (2016). Critical role of redox mediator in suppressing charging instabilities of lithium–oxygen batteries. J. Am. Chem. Soc..

[31] Zhu YG, Wang X, Jia C, Yang J, Wang Q (2016). Redox–mediated ORR and OER reactions: redox flow lithium oxygen batteries enabled with a pair of soluble redox catalysts. ACS Catal..

[32] Zhu YG (2015). Dual redox catalysts for oxygen reduction and evolution reactions: towards a redox flow Li–O_2_ battery. Chem. Commun..

[33] Yao KPC (2016). Utilization of cobalt bis(terpyridine) metal complex as soluble redox mediator in Li–O_2_ batteries. J. Phys. Chem. C.

[34] Pande V, Viswanathan V (2017). Criteria and considerations for the selection of redox mediators in nonaqueous Li–O_2_ batteries. ACS Energy Lett..

[35] Cornut R, Griveau S, Lefrou C (2010). Accuracy study on fitting procedure of kinetics SECM feedback experiments. J. Electroanal. Chem..

[36] Cornut R, Lefrou C (2007). A unified new analytical approximation for negative feedback currents with a microdisk SECM tip. J. Electroanal. Chem..

[37] Taylor AW, Qiu F, Hu J, Licence P, Walsh DA (2008). Heterogeneous electron transfer kinetics at the ionic liquid/metal interface studied using cyclic voltammetry and scanning electrochemical microscopy. J. Phys. Chem. B.

[38] Meini S, Elazari R, Rosenman A, Garsuch A, Aurbach D (2014). The use of redox mediators for enhancing utilization of Li_2_S cathodes for advanced Li–S battery systems. J. Phys. Chem. Lett..

[39] Bergner, B. J. et al. How to improve capacity and cycling stability for next generation Li–O_2_ batteries: approach with a solid electrolyte and elevated redox mediator concentrations. *ACS Appl. Mater. Interfaces* **8**, 7756–7765 (2016). 10.1021/acsami.5b10979 26942895

[40] Gao X, Chen Y, Johnson LR, Jovanov ZP, Bruce PG (2017). A rechargeable lithium–oxygen battery with dual mediators stabilizing the carbon cathode. Nat. Energy.

[41] McCloskey BD, Addison D (2017). A viewpoint on heterogeneous electrocatalysis and redox mediation in nonaqueous Li–O_2_ batteries. ACS Catal..

[42] Mo Y, Ong SP, Ceder G (2011). First–principles study of the oxygen evolution reaction of lithium peroxide in the lithium–air battery. Phys. Rev. B.

[43] Bard, A. J. & Faulkner, L. R. *Electrochemical Methods. Fundamentals and Applications* 2nd edn (Wiley, New York, 2000).

