# Supplementary material for: Kinetics of lithium peroxide oxidation by redox mediators and consequences for the lithium–oxygen cell
Source: Nat Commun. 2018 Feb 22;9:767. doi: 10.1038/s41467-018-03204-0 (PMC5823882; doi:10.1038/s41467-018-03204-0)
Supplement: Supplementary file 1 — Supplementary Information [file 41467_2018_3204_MOESM1_ESM.pdf]

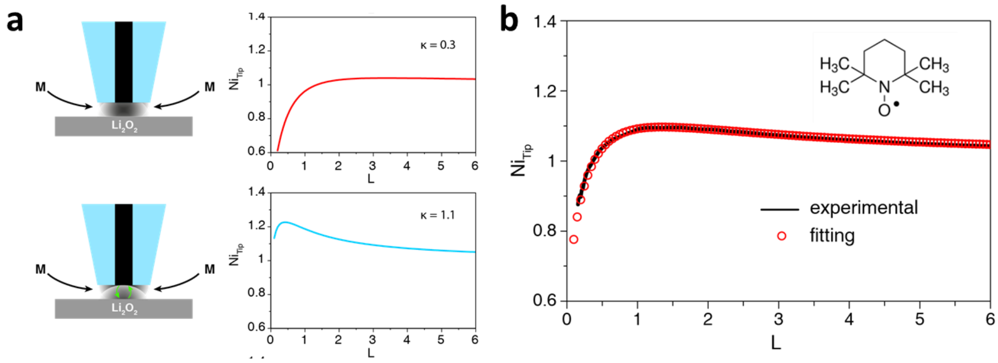

Supplementary Figure 1 | Schematics of feedback approach curves. **a** Schematics of feedback approach curves with various reaction kinetics of mediators oxidizing Li_2O_2. **b** Approach curve (black) and fitting (red circles) of a Au SECM tip towards Li_2O_2 disk in 10mM TEMPO in 100 mM LiTFSI in 4G under Ar. Ni_{tip}, SECM tip current normalized by steady state current; L, dimensionless distance normalised by tip radius; κ, dimensionless rate constant. Radius of electrode was 5 um and NG factor was 3.5.

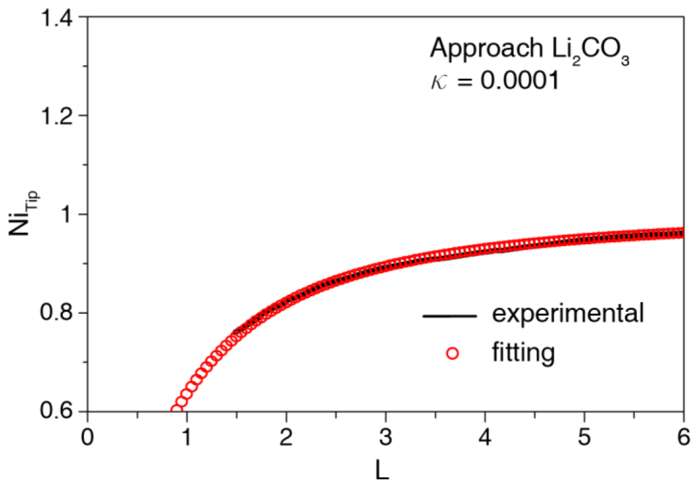

Supplementary Figure 2 | Approach curve and fitting of a Au SECM tip towards Li_2CO_3 disk in 10mM TEMPO in 100 mM LiTFSI in tetraglyme under Ar. The small κ value indicates a slow rate constant of $TEMPO^+$ oxidizing Li_2CO_3. Radius of electrode was 12 um and NG factor was 3.5 k_{app} = 2.4 x 10^{-7} cm s^{-1}.

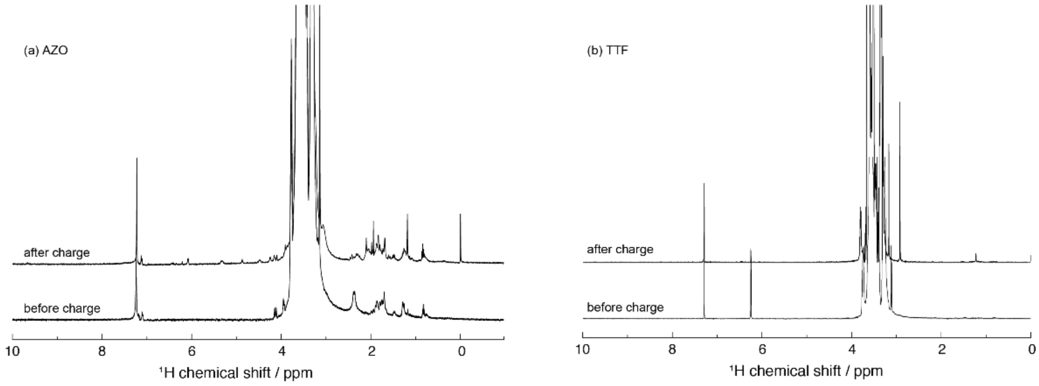

Supplementary Figure 3 | ^{1}HNMR spectra of the TTF-based and AZO-based electrolyte after charging demonstrating the side reaction products. $CDCl_3$ extract from electrodes and separators in cells charged in TTF-based and AZO-based electrolyte in tetraglyme. The peaks marked with arrows are assigned to decomposition products of the RM and the electrolyte. Considerable amounts of mediators were decomposed during the reaction, as indicated by the relative peak areas for mediators and the decomposition product.

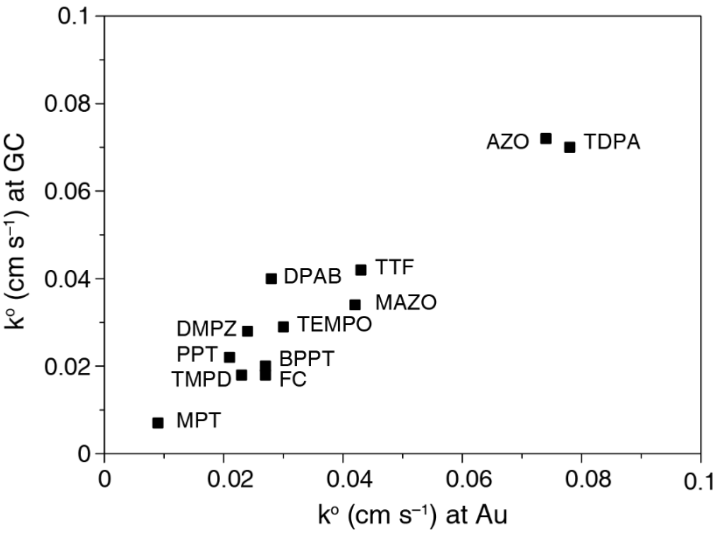

Supplementary Figure 4 | Comparison of k^o measured at a Au electrode and a glassy carbon (GC) electrode. k^o for different redox mediators appears independent of electrode substrate.

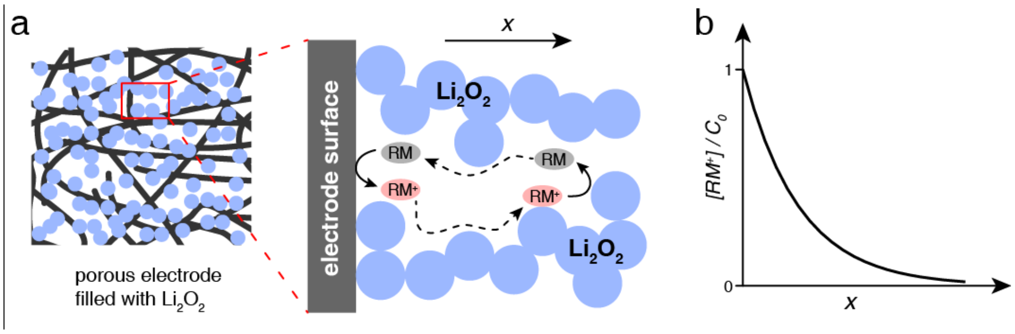

Supplementary Figure 5 | Schematics of porous electrode structure and concentration profile of mediator in oxidized status. **a** Schematics of charging process with redox mediator (RM) at a porous electrode filled with Li_2O_2. Mediator was oxidized at electrode surface, diffused away and oxidized Li_2O_2. **b** The concentration profile of oxidized mediator decays versus the distance away from electrode surface, x.

Supplementary Table 1 Electrochemical properties of various mediators. E^o, standard redox potentials of mediators in 0.1 M LiTFSI in tetraglyme; D, diffusion coefficients; k^o, standard heterogeneous electron transfer rate constants of the reversible redox process; k_{app}, apparent rate constants of various mediators oxidising Li_2O_2.

RM	E^o (V vs. Li^+/Li)	D (10^{-6} cm^2 s^{-1})	k^o (cm s^{-1})	k_{app} (10^{-3} cm s^{-1})
TMPD	3.24	3.6	0.023	0.30
DPAB	3.68	1.2	0.028	0.13
TDPA	3.09	1.5	0.078	0.025
AZO	3.69	3.6	0.074	7.92
MAZO	3.65	2.8	0.042	6.72
TEMPO	3.76	2.9	0.030	3.65
MPT	3.84	3.5	0.007	2.36
PPT	3.88	2.8	0.021	1.82
BPPT	3.84	1.8	0.027	0.82
TTF	3.43	2.8	0.043	0.63
FC	3.51	1.2	0.027	0.22
DMPZ	3.29	2.7	0.024	0.18

Supplementary Notes

The estimated areal current density of the mediator oxidation was calculated based on the Butler–Volmer equation. Assuming a mediator with a 10 mM concentration was oxidised at a porous electrode with a ratio of true surface area to geometric area of 100, an areal current density, *j*, is obtained according to Supplementary Equation 1:

$$j = AFC_0k^oe^{-\alpha F\eta/RT}. \qquad \text{Equation (1)}$$

Herein, *A* is ratio of true surface area to geometric area, *F* is Faraday constant, C_0 is concentration of mediator, k^o is heterogeneous electron transfer rate constant of mediator, α is charge transfer coefficient, 0.5, *R* is gas constant, *T* is temperature and η is overpotential applied. For a mediator having a k^o of 0.007 cm s^{-1}, an areal current density of 210 mA cm^{-2} could be delivered at an overpotential of 60 mV at 25 ^oC, which is sufficient to sustain the charging process.

When calculating the charging current based on the apparent rate constant, k_{app}, for oxidation of Li_2O_2 by the mediator, a one-dimension free diffusion model was used, as shown in Figure S5a. On a planar electrode, Li_2O_2 toroids (400 nm in diameter) stack loosely at a filling ratio of 50 %. The mediator RM is oxidised at the electrode surface to the oxidised form (RM^+), which then diffuses away from surface and reacts with Li_2O_2, oxidising Li_2O_2 and regenerating itself to RM. Due to the fast kinetics for oxidation of the mediator, RM^+ is in excess at the electrode surface, and the concentration decays to nearly zero away from electrode, see Supplementary Figure 5b. Assuming the reaction is in a steady state, all RM^+ being generated at the surface reacts with Li_2O_2 toroids and is consumed, therefore the concentration profile of the RM^+ is constant. The charge current from oxidation of the RM's is equal to the rate of Li_2O_2 decomposition. The current density of oxidation is obtained as Supplementary Equation 2:

$$j = FD'\frac{d(C_0 - C(x))}{dx}|_{x=0} \qquad \text{Equation (2)}$$

Herein *F* is the Faraday constant, *D'* is the effective diffusion coefficient in the porous structure and *C(x)* is the concentration of RM^+ at a distance of *x* away from the electrode surface. As *C(x)* doesn't vary with time in a steady state, in a unit volume,

$$\frac{\partial C}{\partial t} = D'\frac{\partial^2 C}{\partial x^2} - k_{app}CA_{Li2O2} = 0 \qquad \text{Equation (3)}$$

The first term is a second derivative of the concentration profile of RM^+, representing the rate of concentration change caused by diffusion based on Fick's second law and the second term is the rate of reaction between RM^+ and the Li_2O_2 surface. A_{Li2O2} is the surface area of the Li_2O_2 in the unit volume. According to the boundary condition *C(0)*=C_0 and *C(∞)*=0, the concentration profile of RM^+, Supplementary Equation 4, can be worked out from Supplementary Equation 3.

$$C(x) = C_0e^{-\sqrt{\frac{A_{Li2O2}k_{app}}{D'}}x} \qquad \text{Equation (4)}$$

Combining Supplementary Equations 3 and 4, the current density *j* is obtained, Supplementary Equation 5:

$$j = FC_0\sqrt{D'A_{Li2O2}k_{app}} \qquad \text{Equation (5)}$$

In an electrode filled with 400 nm diameter Li_2O_2 toroids at a filling ratio of 50 %, assuming all of Li_2O_2 surface is accessible, A_{Li2O2} is 1 × 10^5 cm^{-1}. For a given C_0 of 0.01 M, k_{app} of 2.5 × 10^{-5} cm s^{-1} and a given effective diffusion coefficient, *D′* of 5 × 10^{-7} cm^2 s^{-1} in the porous structure, it delivers a charging current density of 1.08 mA cm^{-2}. Considering a ratio of true surface area to geometrical surface area of 100 in a porous electrode structure, an areal current density of 108 mA cm^{-2} can be achieved. Based on the same model, a k_{app} of 7.9 × 10^{-3} cm s^{-1} leads to an areal current density of 1.9 A cm^{-2}. This equivalent charging current varies with consumption of Li_2O_2. When Li_2O_2 decomposes, the decrease of the Li_2O_2 surface area and longer diffusion distance to access Li_2O_2 lead to a decrease of the equivalent current, whereas the effective diffusion coefficient *D′* increases and results in an increase of current. The change of this equivalent current depends on which factor is more dominant. At the end of charge, the current decreases dramatically due to the limited availability of the Li_2O_2 surface.
